# Evaluating the Effect of Azole Antifungal Agents on the Stress Response and Nanomechanical Surface Properties of *Ochrobactrum anthropi* Aspcl2.2

**DOI:** 10.3390/molecules25153348

**Published:** 2020-07-23

**Authors:** Amanda Pacholak, Natalia Burlaga, Ewa Kaczorek

**Affiliations:** Institute of Chemical Technology and Engineering, Poznan University of Technology, Berdychowo 4, 60-695 Poznan, Poland; burlaganatalia@gmail.com (N.B.); ewa.kaczorek@put.poznan.pl (E.K.)

**Keywords:** xenobiotics, pharmaceuticals, oxidative stress, environment, hydrophobicity, permeability, roughness

## Abstract

Azole antifungal molecules are broadly used as active ingredients in various products, such as pharmaceuticals and pesticides. This promotes their release into the natural environment. The detailed mechanism of their influence on the biotic components of natural ecosystems remains unexplored. Our research aimed to examine the response of *Ochrobactrum anthropi* AspCl2.2 to the presence of four azole antifungal agents (clotrimazole, fluconazole, climbazole, epoxiconazole). The experiments performed include analysis of the cell metabolic activity, cell membrane permeability, total glutathione level and activity of glutathione S-transferases. These studies allowed for the evaluation of the cells’ oxidative stress response to the presence of azole antifungals. Moreover, changes in the nanomechanical surface properties, including adhesive and elastic features of the cells, were investigated using atomic force microscopy (AFM) and spectrophotometric methods. The results indicate that the azoles promote bacterial oxidative stress. The strongest differences were noted for the cells cultivated with fluconazole. The least toxic effect has been attributed to climbazole. AFM observations unraveled molecular details of bacterial cell texture, structure and surface nanomechanical properties. Antifungals promote the nanoscale modification of the bacterial cell wall. The results presented provided a significant insight into the strategies used by environmental bacterial cells to survive exposures to toxic azole antifungal agents.

## 1. Introduction

**Figure 1:**
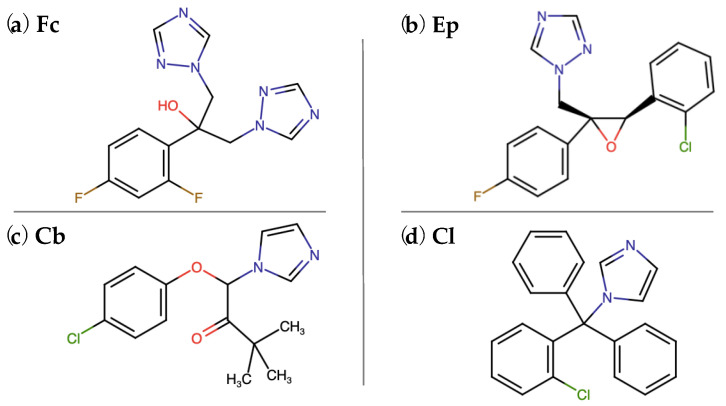
Chemical structures of azole antifungal molecules used in the experiments. Fc—fluconazole, Ep—epoxiconazole, Cb—climbazole, Cl—clotrimazole.

Antibiotics, preparations of plant origin and synthetic drugs are used to combat fungal diseases. Synthetic antifungal agents, a group of numerous organic compounds that differ in structure and mechanism of action, include derivatives of imidazole and triazole. The basic structural unit of all antifungal azoles is a five-membered aromatic ring, which is connected by a nitrogen-carbon bond with other aromatic rings (Figure 1) [1,2]. Azole antifungal molecules are widely used as active antifungal ingredients in various products, such as pharmaceuticals, personal care products and pesticides. Azole antifungals are available as oral formulations, aerosols and ointments. Apart from their applications in pharmacy and medicine, azole fungicides are used as active ingredients of personal care products such as hair shampoos, skin creams, soaps, toothpastes and shower gels [2,3,4]. Clotrimazole, a major topically administered representative of azole antifungal agents, is effective against human fungal infections [4,5]. Clotrimazole is available as cream, spray or solution at concentrations ranging from 1–10% [6]. Fluconazole, a triazole derivative, is particularly active in combating yeast infections. It is taken orally as pills or capsules [7], the minimum effective concentration of fluconazole for antifungal prophylaxis is 0.4 μg mL^−1^ [8]. Another commonly used azole derivative is climbazole. This compound, being an imidazole derivative, is used in the treatment of surface and systemic fungal diseases [2]. The minimum inhibitory concentration of climbazole for fungal species associated with dandruff ranges from 125 to 62.5 μg mL [9].

A broad spectrum of azole fungicides action is not only exploited in the treatment of fungal infections of humans and animals. They are also used in agriculture as the active ingredients of biocides. Triazoles are the most popular class of antifungal agents in agriculture. One of the most commonly used, and characterized by strong antifungal activity is epoxiconazole which is applied in the farmlands at a concentration ranging from 75 to 125 g ha^−1^ [10].

The use of antimicrobial compounds is increasing every year. Unfortunately, many studies have confirmed that these substances are easily introduced into the environment. Active pharmaceutical ingredients have been detected in soil, water, sediments, sludge, and drinking water, often at concentrations remaining at trace levels [11,12,13]. Such micropollutants have been released into the environment by direct discharge of raw or treated municipal and hospital wastewater, industrial sewage treatment plants, surface overflows from agricultural or urban areas [14]. Most wastewater treatment plants (WWTPs) are designed to remove mainly organic nutrients such as organic carbon, nitrogen and phosphorus chemicals. Therefore, many persistent and toxic microcontaminants are ignored. The result is the incomplete degradation of micropollutants in sewage treatment plants and, consequently, the penetration of these compounds and their metabolites into the natural environment [15,16].

The main source of environmental pollution with azole fungicides is domestic sewage, with a partial share of hospital wastewater [17]. The presence of azole pharmaceuticals in treated wastewater has been mainly noted in European countries (Germany, Sweden, Belgium, Switzerland, Ireland, Poland, Spain, Great Britain) and China. Clotrimazole, climbazole and fluconazole are azole derivatives that are often found in treated wastewater in alarming amounts. They can be found not only in the liquid phase, but also in the suspended solid phase. The latter is particularly dangerous as it may become a secondary pollution source of these compounds into the environment [18,19]. For example, fluconazole and clotrimazole were detected in the influent of WWTPs at concentrations up to 109 and 78 ng L^−1^, respectively [20]. Moreover, fluconazole was found in the lake water at concentration up to 9 ng L^−1^ [20] and clotrimazole in river water up to 29 ng L^−1^ [21]. The experiments performed by Peng et al. (2012) revealed the presence of fluconazole, clotrimazole, econazole, ketoconazole, and miconazole in the Chinese wastewater at concentrations ranging from 1–1834 ng L^−1^ [4]. The predicted environmental concentration ranges of climbazole in China in 2015 were 0.009–25.2 ng g^−1^ dry weight in sediments and 0.20–367 ng L^−1^ in water [22].

Although the presence of pharmaceuticals is not always associated with harm to the environment or human health, there are growing concerns about antimicrobial resistance and chronic effects on biodiversity [23]. Residues of azole antifungal agents in the natural ecosystems (especially the aquatic environment) cause many adverse effects on living organisms at many levels of the organization. These micropollutants have a toxic effect on algae growth, they show significant regulation of the expression of drug transporter genes and a change in steroidogenesis regulated by cytochrome P450 [2,24]. Azole fungicides also have a negative effect on the development as well as endocrine and reproductive systems of many organisms. This is particularly evident in fish where many changes occur after contact with azole molecules: masculinization during the development of the gonads, reduced egg production, Leydig cell’s proliferation, and advanced germ cell development [25]. Micropollutants appearing in natural ecosystems cause damage to environmental microorganisms. The specific orientation of pharmaceuticals on biochemical processes and their bioactivity at low concentrations make them a potential ecotoxicological threat [26]. It has been proved that microcontaminants affect the structure and function of biofilms and contribute to a significant decrease in the diversity of microorganisms [27,28]. A great concern is also the fact that some micropollutants do not cause significant toxic effects in a single action, but the combination of different pharmaceuticals can significantly increase their ecotoxicity. The presence of such chemical compounds has a toxic effect on the community of microorganisms, affects cell metabolism and inhibits their growth, which ultimately leads to cells’ death [29]. Another negative effect of pharmaceuticals on the prokaryotic and eukaryotic cells is the accumulation of excessive reactive oxygen species, which results in severe oxidative stress. It causes damage to cell components, such as mitochondria, cell membranes or cell nuclei, as well as associated enzymes, lipids and proteins [30].

The examples presented undoubtedly confirm that the ubiquity of pharmaceutical molecules, including azole antifungal agents, in various environmental compartments, such as soil, groundwater, surface water or wastewater, is a serious environmental problem. Therefore, an important and at the same time demanding task is to examine the impact of these compounds on the cells of environmental bacteria and to remove them from the natural environment.

Among many beneficial environmental bacterial strains, the ones belonging to the *Ochrobactrum* genus are of particular interest to environmental scientists due to their potential application in the bioremediation of many organic and inorganic micropollutants [31,32,33]. The ability of *Ochrobactrum* spp. to reduce chlorate [34], remove polychlorinated biphenyls [35] and toxic metals such as Cd (II) [36] or Cd (III) [31] from the environment has been proved. *Ochrobactrum anthropi*, a Gram-negative, heterotrophic bacterium is abundant in the environment. Various strains of *O. anthropi* have been recently isolated from many environmental compartments globally, for example, activated sludge collected from WWTPs in China [34,37], microbial mats collected from the delta region of the Ebro River [31] or sediment harvested from the surrounding of former chemical factory in Slovakia [35].

Hence, the aim of this research was to examine the response of *Ochrobactrum anthropi* AspCl2.2—a newly isolated bacterial strain—to the presence of the four most common azole antifungal agents (clotrimazole, fluconazole, climbazole, epoxiconazole—Figure 1). Fungicidal derivatives of azole compounds were chosen in such a way to represent particular groups of substances used in various fields of the industry. The experiments performed include the evaluation of the cells’ oxidative stress response as well as changes in the adhesive and elastic properties of the cells. The results have provided a significant insight into the strategies used by environmental bacterial cells to survive exposures to toxic and potentially lethal azole antifungal agents.

## 2. Results

### 2.1. Microbial Stress Response to Azole Antifungal Agents

**Figure 2:**
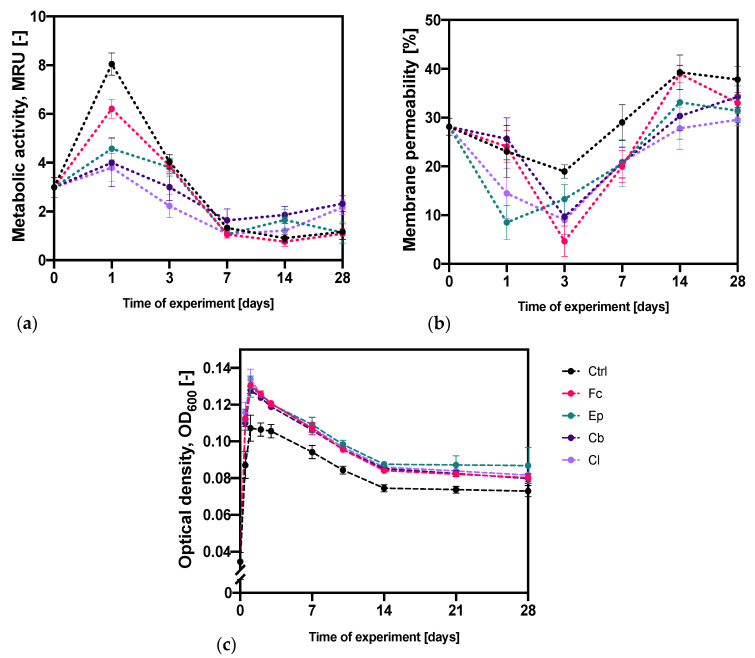
Changes of (**a**) cell metabolic activity; (**b**) cell membrane permeability; (**c**) optical density of bacterial cells exposed to fluconazole (Fc), epoxiconazole (Ep), climbazole (Cb) and clotrimazole (Cl). Ctrl stands for control samples—microbial cultures without the addition of azole molecules. The metabolic activity is defined in MRU (MTT reducing unit). One MRU corresponds to the absorbance of a solution resulting from the dissolution of the formazan crystals formed by mL of cells per OD600. Cell membrane permeability is expressed as a %.

**Figure 3:**
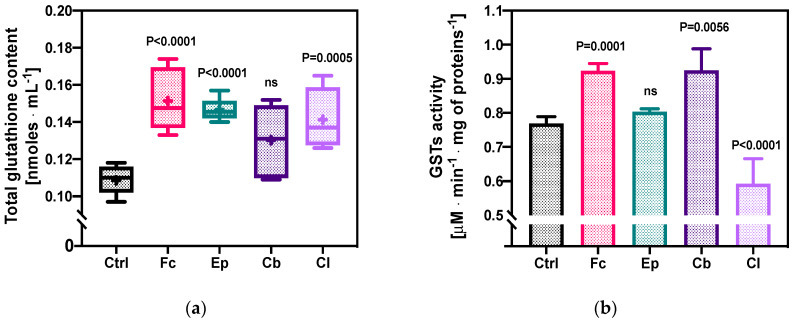
Microbial stress response to the presence of azole antifungal agents: (**a**) the level of total glutathione (oxidized glutathione, GSSG + reduced glutathione, GSH); (**b**) activity of glutathione-S-transferases (GSTs). Bacterial cells were exposed to fluconazole (Fc), epoxiconazole (Ep), climbazole (Cb) and clotrimazole (Cl). Ctrl stands for control samples—microbial cultures without the addition of azole molecules; ns = not significant. See Section 4.7. for the description of statistical analysis.

In order to determine the microbial stress response to the presence of fluconazole (Fl), epoxiconazole (Ep), climbazole (Cb) and clotrimazole (Cl), several techniques were applied. First of all, changes in the cell metabolic activity and cell membrane permeability over a period of 28 days were determined. These experiments were complemented with evaluation of the changes in the total glutathione content (GSSG + GSH) and activity of glutathione-S-transferases (GSTs) of *O. anthropi* AspCl2.2 cells in the exponential growth phase. The results of these experiments are depicted in Figure 2 and Figure 3.

#### 2.1.1. Analysis of the Metabolic Activity and Membrane Permeability

Figure 2a depicts the results of *O. anthropi* AspCl2.2 metabolic activity (MA) measurements and Figure 2c represents the accompanying modifications of cultures optical density. The measurements of optical density (the volume of 0.2 mL) were performed with the use of the microplate’s reader MultiskanSky (Thermo Fisher Scientific, Waltham, MA, USA). The metabolic activity of bacterial cells at the beginning of the experiments had an average value of 2.99 ± 0.41 MRU (MTT reducing unit). After 24 h, the MA significantly increased in all samples and reached the highest value over the entire period of cultivating. Out of all the tests, the greatest MA was obtained for the control sample (8.05 ± 0.46 MRU). The presence of azole derivatives in bacterial culture contributed to a significant reduction in the MA with the lowest value obtained for bacterial cells cultivated with clotrimazole and climbazole. In the third day, the MA of bacterial cells started decreasing; however, it was still higher than at the beginning of the experiments in all cultures except for the one with Cl. The situation has changed after seven days, when the MA has significantly reduced. For the samples with Ep, Cb and Cl, the MA was the lowest with the measured values of 1.06 ± 0.05, 1.64 ± 0.47 and 1.09 ± 0.18 MRU. On the other hand, the control samples and samples with Fc were characterized by the lowest MA in the fourteenth cultivation day. After reaching the lowest metabolic activity, the values began to gradually increase in the following days. Such a relationship was observed in all cultures except for the one with epoxiconazole. As a conclusion, the bacteria cultivated in the presence of Fc, Cb and Cl reflected the tendency of changes in the metabolic activity of bacterial cells that was observed in the control sample. The nature and direction of this parameter modifications were similar; however, the absolute values were mostly lower for bacteria treated with azoles. The rate of the modifications was also different—the cultures with Ep, Cb and Cl reached the lowest MA in day 7 and cultures with Fc as well as control cultures achieved the lowest MA in 14th cultivation day. Surprisingly, the bacteria cultivated in the presence of Cb and Cl were characterized by higher metabolic activity than the reference bacteria at the end of the experiment.

Figure 2b shows modifications of the cell membrane permeability of *O. anthropi* AspCl2.2 over the time of cultivation induced by the presence of azole antifungal agents. In most of the cases, the membrane permeability of cells exposed to azole antifungal molecules was lower than that of control cells. This might have been associated with a defense mechanism triggered in the microbial cells. At the beginning of the experiment, the cells were characterized by an average permeability of 28.1 ± 1.7%. Thereafter, the permeability of the bacterial membrane has reduced in most samples, achieving its lowest value in the first day of cultivation in the presence of Ep and in the third day of cultivation in all other cases. After reaching the lowest permeability of the bacterial membrane, the values began to gradually increase in the following days. The highest permeability was achieved on the 14th day in control samples as well as samples with the addition of Fc and Ep. In samples with Cb and Cl, the highest value of permeability was noted on day 28. The bacterial cultures that contained Fc were the only ones that reflected the tendency of changes in the permeability of the bacterial cell membrane that was observed in the control samples. The direction of changes of this parameter was similar in both cultures; however, the fluctuations of these modifications were stronger in bacteria exposed to Fc.

#### 2.1.2. Total Glutathione Content and GSTs’ Enzymatic Activity

The next stage of the research was devoted to the analysis of changes in the content of the total glutathione (oxidized glutathione, GSSG + reduced glutathione, GSH) in *O. anthropi* AspCl2.2. cells under the influence of azole derivatives (Figure 3a). The control sample, which did not contain azole compounds, was characterized by a glutathione content of 0.109 ± 0.007 nmoles·mL^−1^. In all samples exposed to azoles, the (GSSG + GSH) level in the bacterial cells increased compared to the control sample. The highest increase in glutathione concentration was observed in bacterial cells exposed to Fc and Ep, with the values of 0.151 ± 0.017 and 0.147 ± 0.006 nmoles·mL^−1^, respectively. The smallest increase in the glutathione content in the cells of *O. anthropi* AspCl2.2. was recorded for the culture containing Cb (0.130 ± 0.018 nmoles·mL^−1^). It is also worth noting that the content of GSH in bacterial cells in samples with fluconazole, epoxiconazole and climbazole did not significantly differ from one another.

The analysis of the changes in the activity of GSTs, a group of enzymes that play a key role in the detoxication of many different xenobiotics, is presented in Figure 3b. The enzymatic activity of bacterial cells in the control samples was 0.77 ± 0.01 μM·min^−1^·mg of proteins^−1^. The exposure of bacterial cells to fluconazole and climbazole induced a significant increase in GSTs activity to 0.92 ± 0.02 and 0.89 ± 0.10 μM·min^−1^·mg of proteins^−1^, respectively. Contact of the bacterial strain with Cl reduced glutathione transferase activity to the level of 0.60 ± 0.06 μM·min^−1^·mg of proteins^−1^ (*p* < 0.0001). The only compound that did not modify the GSTs’ enzymatic activity of *O. anthropi* AspCl2.2. was epoxiconazole.

### 2.2. Bacterial Cell Topography and Surface Roughness Determination

**Figure 4:**
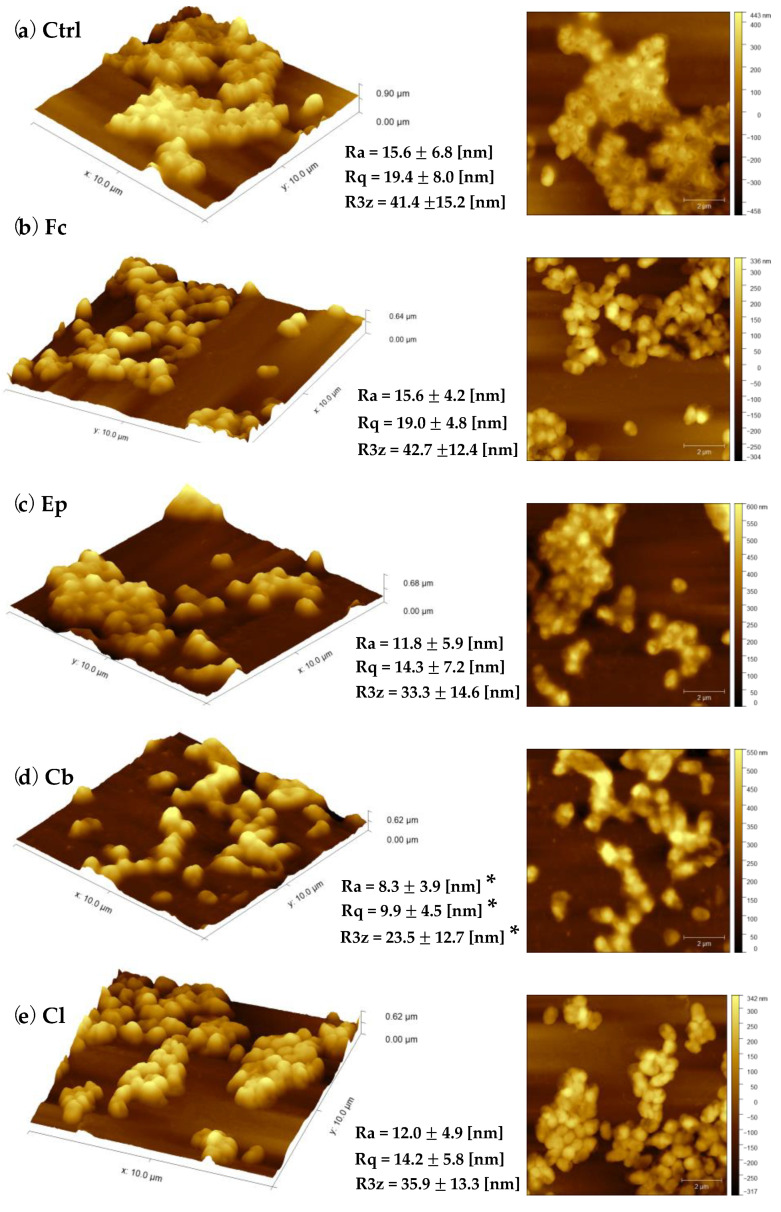
Representative atomic force microscopy (AFM) three-dimensional and height images supplemented with surface roughness parameters (mean value ± SD) showing morphological differences between (**a**) untreated control cells of *O. anthropi* AspCl2.2; cells exposed to: (**b**) fluconazole; (**c**) epoxiconazole; (**d**) climbazole; (**e**) clotrimazole. Asterisks (*) indicate a statistically significant difference (Cb vs. Ctrl: Rq *p* = 0.0046; R3z *p* = 0.0226; Ra = 0.0124). See Figure S1 for the bacterial AFM images of a larger scale.

Texture and morphology of *O. anthropi* AspCl2.2. cells taken from the 3-day liquid cultures have been measured using an atomic force microscopy (Park NX10, Park Systems Corp., Suwon, Korea). Visualization of porous morphology and the nanoscale structure of cell surface have been made for bacteria before and after exposure to azole antifungal agents (Figure 4 and Figure S1). Bacterial cells were imaged in air with the use of non-contact mode. Figure 4a shows typical, representative 10 × 10 μm atomic force microscopy (AFM) three-dimensional and height images of untreated *O. anthropi* AspCl2.2 cells. The surfaces of the cells were topographically homogenous, and the average dimensions of the cells were as follows: length 0.62 ± 0.08 μm; width 0.34 ± 0.06 (Table 1). The cells were mostly packed close to one another and exhibited a tendency towards aggregation. A light boundary around the cells aggregates indicates that the control cells were coated with an extracellular substance.

The exposure of bacterial cells to azole fungicides resulted in increased cells’ dimensions in all samples (Table 1). When compared the cells exposed to azole antifungals with control cells, they looked to be inflated. Among all bacterial cells exposed to azole fungicides, the ones exposed clotrimazole and fluconazole seemed to be the most homogenous, and their surface was the smoothest. It can be also seen that the bacteria cultivated under stress conditions did not have such a strong tendency towards aggregation as the control cells.

Analysis of the changes in cell topography was accompanied by calculation of bacterial cell surface roughness. Among all compounds tested, climbazole was the only one that affected all surface roughness parameters. An average roughness depth (R3z), root mean square roughness (Rq) and roughness average (Ra) of cells exposed to climbazole decreased significantly in comparison to control cells. The texture of cells exposed fluconazole, epoxiconazole and clotrimazole has not been affected.

### 2.3. Microbial Cell Surface Properties

**Figure 5:**
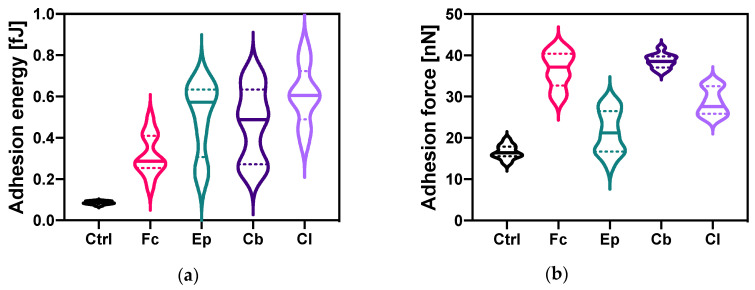

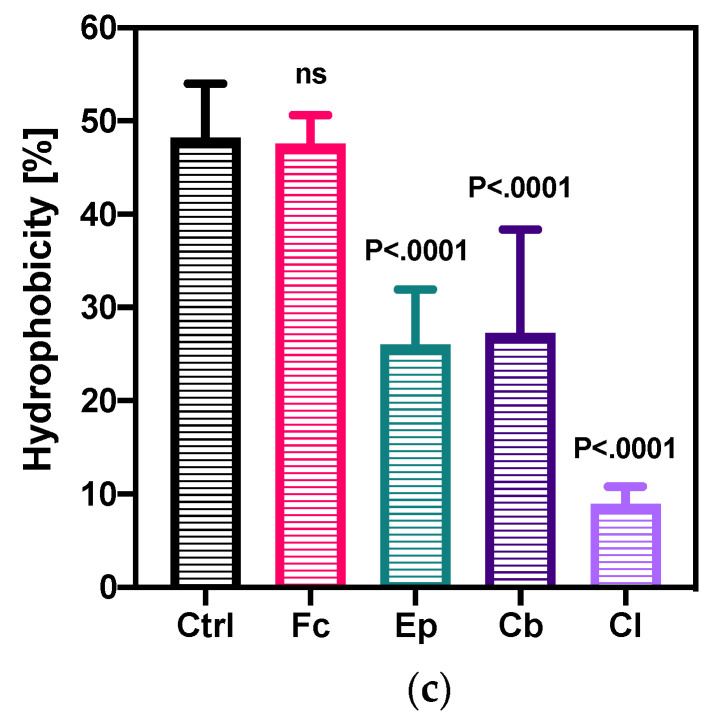
Bacterial cell adhesive properties: (**a**) adhesion energy; (**b**) adhesion force; (**c**) cell surface hydrophobicity. In violin plots, the difference between each of the treated samples and control sample was statistically significant (*p* < 0.0001); ns = not significant. See Section 4.7 for the description of statistical analysis.

**Figure 6:**
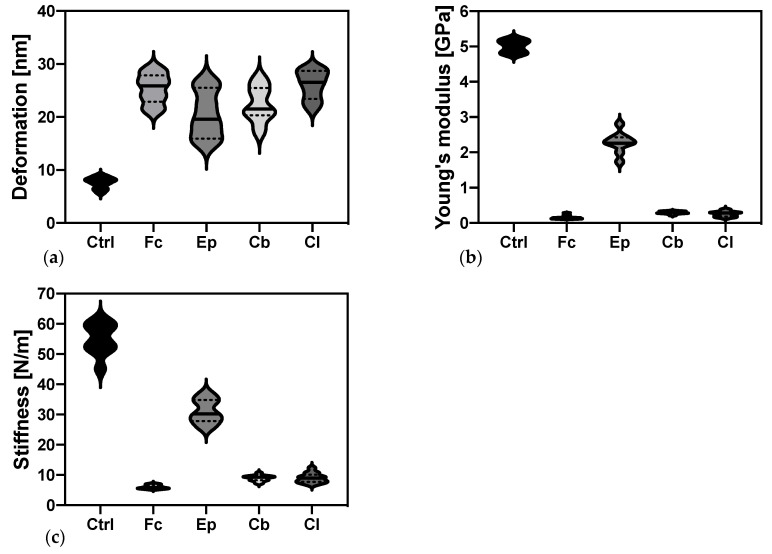
Empirical distribution of the data of bacterial cell elastic properties measurements: (**a**) changes in bacterial cell wall deformation; (**b**) Young’s modulus; (**c**) stiffness. The difference between each of the treated samples and control sample was statistically significant (*p* < 0.0001). See Section 4.7 for the description of statistical analysis.

The next stage of our research involved analysis of alterations in microbial cell surface properties, indicating variations in cell adhesion and elasticity. The investigation of azole antifungal molecules’ effects on bacterial cell adhesive properties included the combination of two different techniques. The first one was based on the measurements of bacterial cells’ adhesion force and adhesion energy (Figure 5a,b). The second method involved evaluation of the cell surface hydrophobicity by spectrophotometric measurements of Congo Red adsorption on the surface of the cells (Figure 5c). Moreover, bacterial cell elastic properties were measured and calculated using atomic force microscopy and PinPoint™ Nanomechanical Mode (Figure 6).

#### 2.3.1. Changes in The Bacterial Cell Adhesion

Alterations of adhesion energy due to the presence of azole derivatives in the bacterial culture have been quantified with nanoscale resolution and are presented in Figure 5a. The cell adhesion energy in the control samples was 0.085 ± 0.008 fJ. A statistically significant increase in the energy of cell adhesion of bacteria under the influence of each of the azoles was observed, compared to the control sample. Exposure of bacteria to clotrimazole increased this parameter, to a value of 0.61 ± 0.14 fJ. A similar result was obtained after bacterial contact with epoxiconazole (0.57 ± 0.18 fJ). The smallest increase in the adhesion energy of *O. anthropi* AspCl2.2. cells (from 0.085 ± 0.008 fJ to 0.29 ± 0.10 fJ) compared to control sample was obtained for fluconazole.

Figure 5b depicts modifications of the adhesion force of *O. anthropi* AspCl2.2. cells upon contact with azole compounds. The value of this parameter for the control sample was 16.6 ± 1.7 nN. The addition of azole antifungal agents to bacterial cultures resulted in increased cells adhesion force (statistically significant difference between control sample and treated sample was noted for each bacterial culture). The greatest change in adhesion force was recorded for samples with climbazole and fluconazole, reaching the values of 38.6 ± 1.7 and 37.2 ± 4.3 nN, respectively. The smallest increase in the adhesion force of the bacterial cells cultivated under stress conditions in comparison with the control sample was noted for the cultures containing epoxiconazole.

The results of *O. anthropi* AspCl2.2 cell surface hydrophobicity measurements are shown in Figure 5c. The hydrophobicity of the control sample was 48.2 ± 5.5%. The exposure of cells to epoxiconazole, climbazole and clotrimazole resulted in a reduction of surface hydrophobicity. The lowest hydrophobicity (8.92 ± 1.79%) was recorded for the cells cultivated in the presence of clotrimazole. The hydrophobicity of bacterial cells in samples containing epoxiconazole and climbazole reached a similar average level of 26.7%. This parameter measured in *O. anthropi* AspCl2.2 cells exposed to fluconazole was similar to that of control cells.

#### 2.3.2. Elastic Properties of *O. anthropi* AspCl2.2 Determined by Atomic Force Microscopy

Atomic force microscopy has been used to quantify the elastic properties of the bacterial cell wall. Alterations of cells elastic modulus, stiffness and deformation upon exposure to azole antifungal agents have been analyzed with nanoscale resolution and the results are presented in Figure 6.

Figure 6a shows changes in the nanomechanics of *O. anthropi* AspCl2.2 deformation measured by AFM. The value of this parameter for the control sample was 7.94 ± 1.02 nm. A statistically significant increase in the deformation of bacterial cells induced by the presence of azole antifungal agents was observed, compared to the control sample. The highest values, 26.5 ± 2.07 and 25.9 ± 2.8 nm, were obtained for the cells cultivated in the presence of clotrimazole and fluconazole. However, the smallest increase in the cells’ deformation was noted for culture cultivated with epoxiconazole.

Elastic modulus as high as 5.00 ± 0.17 GPa was quantified in the reference bacterial cells (Figure 6b). The presence of azole antifungals in the microbial cultures significantly decreased Young’s modulus of bacterial cells compared to the control sample. The smallest decrease (to 2.28 ± 0.30 GPa) was observed for the cells cultivated in the presence of epoxiconazole. In the case of climbazole and clotrimazole, the decrease was already significant, and the smallest value of Young’s modulus of bacterial cells (0.15 ± 0.05 GPa) was recorded in the sample containing fluconazole.

Together with elastic modulus and deformation, changes in bacterial cells stiffness upon addition azole antifungal agents were quantified. Stiffness of control *O. anthropi* AspCl2.2 cells 54.9 ± 5.2 N m^−1^. When the cells were cultivated in the presence of azole antifungals, their cell wall becomes more flexible. The smallest decrease in cell wall stiffness was observed for cells cultivated with epoxiconazole; however, the change was still statistically significant. The lowest stiffness (5.87 ± 0.64 N m^−1^) was recorded for the cells cultivated in the presence of fluconazole.

## 3. Discussion

Throughout the last few decades, the occurrence of emerging contaminants in the environment has attracted the attention of scientists from around the world, due to their potential undesirable long-term ecological effects. It has been demonstrated that the residual amounts of pharmaceuticals, pesticides endocrine disrupting chemicals and personal care products occur at relatively high concentrations in rivers, lakes, groundwater and even tap water or wastewater effluents [3,38,39]. One of the most common chemical compounds, classified as emerging environmental contaminants, are azole fungicides. Their presence in the various environmental compartments has been documented in a number of reports [2,40]. The chronic exposition of azole antifungal compounds to non-targeted organisms is considered to cause negative effects; however, the detailed mechanism of their influence on biotic components of natural ecosystems has remained unexplored. Therefore, in our research, we isolated novel environmental strain *O. anthropi* AspCl2.2 and examined its remodeling of the cell wall and oxidative stress response to the presence of four most commonly used azole fungicides: fluconazole, epoxiconazole, climbazole and clotrimazole. Such an approach allowed for investigating the strategies used by environmental bacterial cells to survive exposures to potentially toxic and lethal azole antifungal agents.

The first part of our research was devoted to analysis of the bacterial oxidative stress induced by the presence of azole antifungals. At the beginning, dynamic changes in the cell metabolic activity (as an indicator of cell viability, proliferation and cytotoxicity) were evaluated using MTT assay, which was based on the conversion of 3-[4,5-dimethylthiazol-2-yl]-2,5 diphenyl tetrazolium bromide into formazan crystals by living cells. Since oxidative stress is known to promote membrane disruption [41], changes in membrane permeability in different time points were also measured (Figure 2). These experiments were complemented with the determination of the changes in the total glutathione content (as an indicator of the overall health of a cell) and the activity of glutathione-S-transferases (enzymes which play a key role in stress response and detoxification) of the cells in the exponential growth phase (Figure 3).

Our results suggest that bacteria triggered an immediate response to the presence of potentially toxic azole antifungals since the strongest differences between the control sample and the azole samples were noted in the first time point measured (24 h). The smallest differences between control and treated bacteria were measured in 7-day cultures. In general, the cell metabolic activity and cell membrane permeability have been changing with time substantially in both the control samples and the samples with azole antifungals. However, these samples differed substantially with the strength of these modifications. In most cases, at the beginning of the experiments, the metabolic activity increased in all cultures; however, after reaching the maximum value, the number of metabolically active cells started decreasing, and after that it began to slowly grow again. Opposite modifications were noted for cellular membrane permeability; at the beginning of the experiment, the bacterial membrane became less permeable, and, after that, a significant increase was noted followed by a reduction in this parameter. These modifications were observed at different rates for each fungicide. Despite the fact that the general trends were similar in control and treated samples, the fluctuations were greater in bacteria treated with azoles. We speculate that such modifications of membrane permeability and cell metabolic activity were triggered by cellular adaptation to the presence of azole antifungal agents. The observations of changes in cellular metabolic activity are in good agreement with previous articles, which described the toxicity of different kinds of xenobiotics on bacterial cells. In a research carried out by Bergheim et al. (2015) [42], bacteria from the *P. putida* strain were exposed to various antibiotics and artificial sweeteners. As it turned out, the high toxicity of the compounds was associated with the low ability of bacteria to degrade these compounds. In another study conducted by Zhang et al. (2013) [43], the effect of furazolidone on *A. calcoaceticus*, *P. putida* and *P. mirabilis* was analyzed. It has been shown that the degradation of this compound has resulted in much less cytotoxic metabolites than the parent compounds. The analysis of the toxicity of aromatic hydrocarbons on *E. coli* and *P. fluorescens* cells performed by Oberoi and Philip (2017) [44] showed that these compounds significantly reduced the metabolic activity of the cells of all strains tested.

Our previous research [5] has shown that contact of bacteria isolated from activated sludge with clotrimazole caused an increase in cell membrane permeability. Contrastingly, Smułek et al. (2019) have observed that *P. plecoglossicida* cells under the influence of 1-chlorotoluene significantly reduced cell membrane permeability [45]. This indicates that the properties of the bacterial cell membrane are different for different species, which results from bacterial adaptation to specific environments [46].

Glutathione, a tripeptide (γ-glutamyl-cysteinylglycine), is one of the most important free thiols in the majority of living cells. It’s involvement in many biological processes, taking place in eukaryotic cells, such as of xenobiotics neutralization, the removal of hydroperoxides, and the maintenance of the cell homeostasis and oxidation state has been well documented [47]. However, the level of glutathione, its transport in bacterial cells as well as its role in bacterial physiology remains poorly investigated [48].

It has been demonstrated that stress factors, such as hyperosmotic shock, treatment with antibiotics or temperature shock, often cause substantial changes in the level of glutathione [48]. Moreover, as GSTs’ enzymes are involved in a defensive mechanism against xenobiotics, their activity might be expected to increase after exposure to toxic substances [49]. The results of quantifications of the total level of glutathione of *O. anthropi* AspCl2.2 cells show a significant increase in the extracellular total glutathione content of bacteria exposed to Fc, Ep and Cl. However, the modifications of the total glutathione content in response to the presence of azole antifungals were not always consistent with alterations in the activity of GSTs enzymes. For example, in the sample containing Cb, the modification of GSH + GSSG was not significant, but a substantial increase in the activity of GSTs was measured. On the contrary, in the cells from the culture containing Ep, enhanced glutathione content was not accompanied by the change in GSTs’ activity. This might indicate that bacteria activated different (and not always complete) defensive and detoxification mechanisms against azole fungicides mentioned.

Another part of our experiments was devoted to the analysis of changes in the morphology of the bacterial cells after exposure to selected azoles. The results indicate that azole antifungals induced significant modifications of structure and shape of *O. anthropi* AspCl2.2 cells. Surprisingly, the presence of all compounds tested except for Cb did not modify bacterial cell texture (Figure 4)—no significant difference between cells exposed to Fc, Ep and Cl and control cells was observed. The exposure of bacterial cells to Cb induced a decrease in all calculated surface roughness parameters. These results were rather unexpected because, more often than not, xenobiotics promote increase in bacterial cell roughness [50]. It has been demonstrated that increased values of microbial cell surface roughness may be associated to the cell membrane damage [51]. This means that decreased surface roughness (as observed in the culture with Cb) may originate from the tightening of bacterial cell membrane in response to a stressor as a part of an adaptation or defensive process. Such interpretation is consistent with the results of cell metabolic activity (Figure 2a), which indicate that the culture containing climbazole contained the highest number of viable cells.

Measurements of microbial cell adhesive and elastic properties allowed for the analysis of changes occurring in the surface of the microbial cell upon the addition of azole antifungal agents. Studies at nanoscale have provided precise structural information about the interaction of bacterial cell and azole fungicides as well as enabled detailed studies of bacterial response to the presence of such xenobiotics as azole fungicides.

Investigation of bacterial adhesive properties is important since these features affect cells metabolic activity and physiology. Regarding adhesion properties, adhesion energy of the cells taken from each culture containing azole fungicide was significantly higher than that of control cells. Importantly, the cells taken from cultures treated with azoles exhibited considerable heterogenicity, which is shown by a wide interquartile range (Figure 5a). Such a phenomenon was not observed for the control cells. Regarding adhesion force, a significant increase in this parameter was also observed. Modifications of the changes in the cell surface hydrophobicity of the cells treated with azoles were opposite to those of adhesion force in samples treated with Ep, Cb and Cl. Comparison of the modifications in adhesive properties measured with these two techniques is important. Traditional classic methods have provided average information about bacterial cells population; however, they lack information on the single bacterial cell level [52,53]. Such information was obtained by AFM measurements. In general, bacterial cells treated with azole antifungal agents exhibited higher adhesive properties than untreated cells, which is expressed by increased adhesive parameters. The highest adhesive properties might be attributed to the cells cultivated in the presence of Fc. We suppose that the cells with higher adhesive properties showed higher expression of adhesins—proteins attached to bacterial cell walls mediating adhesion of the cells [53].

Bacterial cells’ deformations, Young’s moduli and stiffness were determined in single cell experiments using AFM (Figure 6). The nanomechanics of bacterial cells’ deformation revealed molecular defects due to increased parameter in treated samples in comparison to control sample. Opposite modifications were noted for elastic modulus and stiffness. The highest values were noted for the control samples. The cultivation of bacterial cells under stress conditions softened the surface of bacterial cells and decreased Young’s modulus. Similar modifications were observed by Francius et al. (2008) [54]. Such modifications may be related to the changes in peptidoglycan, a key cell surface component which imparts cell mechanical properties, present on the surface of the cell.

## 4. Materials and Methods

### 4.1. Chemicals

Fluconazole (Fl), epoxyconazole (Ep), climbazole (Cb), clotrimazole (Cl) and all other chemicals were obtained from Sigma-Aldrich (Sigma Aldrich, St. Louis, MO, USA), microbiological media were purchased from bioMérieux (Warsaw, Poland). All chemical reagents and solvents were of the highest analytical grade and used without further purification. Solutions used in the experiments were prepared using ultra-pure deionized Milli-Q water (Arium^®^ Pro, Sartorius, Kostrzyn Wlkp., Poland).

Laboratory glassware and all aqueous solutions and were autoclaved before using them in the experiments. Solutions not amenable to autoclaving were filtered through disposable 0.22-μm membrane filters (Captiva EconoFilters, Agilent, CA, USA). A biological safety cabinet (Labculture^®^ Class II, Esco, Singapore) was used during activities associated with biological samples in order to prevent contamination.

### 4.2. Bacterial Strain and Culture Conditions

Activated sludge was collected aseptically from the urban wastewater treatment plant in Poland (52°25′53.1″ N, 16°57′31.8″ E) and used for bacterial strains isolation. The isolation process was carried out as described previously with the mixture of Fl, Ep, Cb and Cl used as a selective agent [55]. The isolated bacterial strain that was able to grow in the presence of azole derivatives was identified using biochemical (Vitek^®^ 2 system, bioMérieux, Warsaw, Poland) and molecular techniques as *O. anthropi* AspCl 2.2 (GenBank: MK503652.1) [56]. The strain isolated was stored in 50% glycerol solution at −20 °C. In order to start the bacterial cultures, cells were placed on the Mueller-Hinton agar plates (bioMérieux, Warsaw, Poland) and incubated for 24 h at 30 °C. Next, a loop full of cells was introduced to a nutrient broth and the bacteria were allowed to grow overnight at 30 °C with shaking (120 rpm, KS 400 ic control, IKA Werke, GmbH, Staufen, Germany). The bacterial suspension obtained was washed three times (4000× *g*, 10 min, Centrifuge 5910R, Eppendorf, Germany) with sterile mineral salt medium (MSM, composition as in [55]). In the final stage, the cell pellet was resuspended in MSM to adjust optical density to 1.0 ± 0.1 at λ = 600 nm (Multiskan Sky, Thermo Fisher Scientific, Waltham, MA, USA). Such prepared cell suspension was used to inoculate bacterial cultures. The cultures were carried out in sterile 250 mL DURAN^®^ laboratory bottles containing 5 mL of inoculum, 45 mL of MSM supplemented with microelements solution, 0.1 mL of 20% sodium succinate and methanolic solution of a given azole derivative (final concentration in the cultures: 0.1 mg L^−1^). The cultures were incubated in 30 °C with shaking.

### 4.3. Analysis of the Metabolic Activity, Total Glutathione and GSTs

In order to analyze the impact of four azole antifungals agents on the cell metabolic activity, the level of total glutathione (GSSG + GSH) and activity of glutathione S-transferases, *O. anthropi* AspCl2.2. cultures containing Fl, Ep, Cb or Cl were established as described in Section 4.2. Concurrently, the reference cultures (Ctrl) without the addition of xenobiotics were carried out.

Microbial cells’ metabolic activity was measured after 1, 3, 7, 14 and 28 days of bacterial exposure to azole derivatives using 3-(4,5-dimethylthiazol-2-yl)-2,5-diphenyltetrazolium bromide (MTT) assay [57]. Briefly, 200 μL of microbial cells suspension was transferred into the sample wells of the sterile microplate. After OD600 of the cultures’ measurements, the cells were collected by centrifugation and resuspended in the same volume of MSM. Next, MTT solution (5 mg mL^−1^ solution in Dulbecco’s Phosphate Buffered Saline (DPBS)) was added to the wells using multichannel pipette and the mixtures were incubated for 2 h in 30 °C in the dark with shaking on an orbital shaker. Later on, the cells were centrifuged (4500× *g*, 30 min, 20 °C, Centrifuge 5910R, Eppendorf, Germany), supernatants were carefully discarded, and the remaining cell pellets were suspended in dimethyl sulfoxide (200 μL). Following a 15-min incubation, the microplates were centrifuged again (4000× *g*, 15 min). After that, 150 μL of supernatant from each well was transferred to a new microplate and absorbance at 560 nm was measured. The higher absorbance of the solution indicated the greater number of viable, metabolically active cells in the sample well. The metabolic activity is defined in MTT reducing units (MRU) and was calculated according to Equation (1):

(1) $$MRU=\frac{A_{560}\cdot SV}{TV}\cdot \frac{K}{OD_{600}}\;\left[-\right]$$

where: $A_{560}$—absorbance of the formazan dissolved in DMSO solution; SV—the volume of the test sample [mL]; TV—total volume of the reaction mixture [mL]; K—dilution factor; $OD_{600}$—optical density of the bacterial cells collected from the cultures.

Abiotic samples, control samples (bacteria without treatment) and treated samples were prepared identically.

The next part of the experiments included analysis of the total glutathione and glutathione S- transferases. Before each experiment, the cells were washed with sterile Dulbecco’s Phosphate Buffered Saline (DPBS) and concentrated to reach optical density *OD*_600_ = 1.0 ± 0.1. The level of total glutathione was examined in deproteinized cell extracts using a Glutathione Assay Kit (Sigma Aldrich, St. Louis, MO, USA) according to the manufacturer’s protocol. The cells were lysed with a freeze–thaw lysis method (liquid nitrogen was used to freeze and a 37 °C bath to thaw).

The measurements of total GSTs activity were performed with the use of a Glutathione S-Transferase (GST) Assay Kit (Sigma Aldrich, St. Louis, MO, USA) according to the manufacturer’s protocol. The cells were lysed using a CelLytic™ B Plus Kit (Sigma Aldrich, St. Louis, MO, USA). The content of proteins was determined with the use of Pierce™ BCA Protein Assay. (Thermo Fisher Scientific, Agawam, MA, USA). The activity of GSTs was calculated and expressed as the μM of product formed by an enzyme in one minute at 30 °C per milligram of total proteins.

### 4.4. Preparation of Bacterial Samples for AFM Analysis and Bacterial Cells’ Surface Characterization by AFM

Treated and control *O. anthropi* AspCl 2.2 cells taken from 3-day liquid cultures were washed three times with sterile DPBS. The pellets were gently resuspended in DPBS to adjust cells’ concentration of 1 · 10^8^ cfu mL^−1^. Subsequently, bacterial suspensions were placed on a freshly cleaved mica surface and allowed to dry in air at room temperature for 2 h before imaging. The mica was attached to a steel puck using double-sided adhesive tape and then transferred onto the sample stage of atomic force microscope (Park NX10, Park Systems Corp., Suwon, Korea).

First of all, the surface plots were made to provide a three-dimensional perspective of the cells’ surface, from which modifications of the bacterial surface were determined and cell roughness parameters were calculated. Measurements were performed using a non-contact mode using an All-in-One cantilever, type D (BudgetSensors, Sofia, Bulgaria) with nominal resonance frequency of 350 kHz and nominal force constant of 40 N/m. The scan size was set to 10 × 10 μm^2^ with sampling of 512 lines for each image and a scan rate of 0.4–0.5 Hz. For each sample, at least three measurements of 10 × 10 μm^2^ area were conducted in different positions in order to localize the bacterial cells and choose the best view of sample positioning. The measurements were carried out in air at room temperature. The results of imaging were further analyzed by Gwyddion software.

Surface roughness was calculated from the single cell images of the same sample. At least fifteen cells of each culture were included in the calculations. An average roughness depth (R3z), root mean square roughness (Rq) and roughness average (Ra) were chosen as indicative of bacterial cell surface alterations. The procedure of roughness calculation is available in Supplementary material 1.

### 4.5. Nanomachanics of Bacterial Cells

Evaluation of changes in selected quantitative nanomechanical properties of bacterial cells (adhesion energy, adhesion force, deformation, stiffness, elastic modulus) was performed using PinPoint™ Nanomechanical Mode from Park Systems. For each experimental setup, at least fifteen cells were included in calculations. The final results are expressed as the average of the replicates and the error bars indicate a 95% CI.

### 4.6. Analysis of Cell Surface Hydrophobicity and Cell Surface Permeability

For the analysis of biochemical cell surface properties, microbial cultures with and without the addition of azole derivatives were established as described in Section 4.2. After 3 days of cultivating, the cells in the mid-log growth phase were centrifuged (4000× *g*, 15 min) and washed with DPBS. Afterwards, they were concentrated to reach optical density OD_600_ = 1.0 ± 0.1. The membrane permeability and cell surface hydrophobicity were measured by measuring the uptake of crystal violet solution by the cells and adsorption of Congo red dye on the surface of the cells [58,59].

### 4.7. Statisticl Analysis

All of the results presented have been obtained from measurements of three independent experiments (biological replicates). For each biological replicate, three technical repetitions were performed. A statistical significance of differences between the means of the samples was determined by one-way analysis of variance (ANOVA). Tukey’s test was used for multiple comparisons. The significance level was established as 5%. P values calculated report up to four digits after the decimal point and are described in the graphs. In the box and whiskers plots, whiskers represent the 10th to 90th percentiles; the box extends from the 25th to 75th percentiles; midline is plotted at the median, “+” is at the mean value. In the bar plots, error bars indicate a 95% CI and the bars represent the median value. In the violin plots, representing the empirical distribution of the data, midline is plotted at the median and dotted lines represent quartiles (Q1 and Q3). The calculations were performed using GraphPad Prism (GraphPad Software, LLC, San Diego, CA, USA).

## 5. Conclusions

The experiments performed indicate that the presence of azole antifungal compounds increases bacterial oxidative stress. The strongest differences were noted for the cells cultivated in the presence of fluconazole. The least toxic effect can be attributed to climbazole (due to its lowest modification of bacterial metabolic activity, bacterial shape and glutathione content). AFM observations unraveled molecular details of bacterial cell texture, structure and surface nanomechanical properties. Based on the results obtained, it can be clearly stated that azole antifungals promote the nanoscale modification of the bacterial cell wall. These changes, taken together with modifications of the cell surface hydrophobicity and membrane permeability have been strictly associated with the remodeling of the cell wall structure and its chemical composition. The results of the different modifications of cell metabolic activity, cell membrane permeability as well as the level of glutathione and GSTs’ activity caused by the four azole antifungals are indicative of different mechanisms of action of the compounds studied. This is in good agreement with atomic force microscopy data that showed that cells were not uniformly affected by azole antifungals. A summarizing table showing the effects of each of the azoles on measured cell parameters is available in Table S1. The results presented have provided a significant insight into the strategies used by environmental bacterial cells to survive exposures to toxic and potentially lethal azole antifungal agents. They clearly indicate that azole antifungal residues may be harmful for environmental bacterial strains.

## Figures and Tables

**Table 1:** Dimensions of individual *O. anthropi* AspCl2.2. cells calculated from AFM images. The results are presented as the mean value ± standard deviation.

	Ctrl	Fc	Ep	Cb	Cl
Cell length [μm]	0.62 ± 0.08	0.75 ± 0.08 *	0.71 ± 0.16	0.76 ± 0.13 *	0.78 ± 0.10 *
Cell width [μm]	0.34 ± 0.06	0.43 ± 0.06	0.49 ± 0.12 *	0.47 ± 0.04 *	0.37 ± 0.06

* Asterisks indicate a statistically significant difference (treated cells vs. control cells) calculated using one-way ANOVA.

## Supplementary Materials

The following are available online at https://www.mdpi.com/1420-3049/25/15/3348/s1, Figure S1: Representative AFM height images showing morphological differences between (a) untreated control cells of O. anthropi AspCl2.2; cells exposed to: (b) fluconazole; (c) epoxiconazole; (d) climbazole; (e) clotrimazole. Table S1: Summarizing table - the effects of each of the azoles on measured cell parameters.
