# Supplementary material for: Evaluating the Effect of Azole Antifungal Agents on the Stress Response and Nanomechanical Surface Properties of Ochrobactrum anthropi Aspcl2.2
Source: Molecules. 2020 Jul 23;25(15):3348. doi: 10.3390/molecules25153348 (PMC7435821; doi:10.3390/molecules25153348)
Supplement: Supplementary file 1 [file molecules-25-03348-s001.zip › Table S1.pdf]

Table S1. Summarizing table - the effects of each of the azoles on measured cell parameters.

	Parameter	Fc	Ep	Cb	Cl
**Oxidative stress response**	GSSG + GSH	↑↑	↑↑	ns	↑↑
	GSTs	↑↑	ns	↑↑	↓
**Cell texture**	Ra	ns	ns	↓↓	ns
	Rq	ns	ns	↓↓	ns
	R3z	ns	ns	↓↓	ns
**Cell adhesion**	Adhesion energy	↑↑↑↑	↑↑↑↑	↑↑↑↑	↑↑↑↑
	Adhesion force	↑↑↑	↑↑	↑↑↑	↑↑
	Cell surface hydrophobicity	ns	↓↓	↓↓	↓↓↓↓
**Elastic properties**	Deformation	↑↑↑↑	↑↑↑	↑↑↑	↑↑↑↑
	Young's modulus	↓↓↓↓	↓↓↓	↓↓↓↓	↓↓↓↓
	Stiffness	↓↓↓↓	↓↓	↓↓↓↓	↓↓↓↓

GSSG + GSH – the level of total glutathione; GSTs – activity of glutathione S-transferases;
Ra – roughness average; Rq – root mean square roughness; R3z – average roughness depth.

- **ns** – the modification was not significant (vs. control sample)
- the direction of the arrows indicates up-/down-regulation
- the number of the arrows indicates the strengths of the modification:

  I – increase/decrease by up to 1.0-fold (vs. control sample)
  II – increase/decrease within the range from 1.1 to 2.0-fold (vs. control sample)
  III – increase/decrease within the range from 2.1 to 3.0-fold (vs. control sample)
  IIII – increase/decrease by at least 3.1-fold (vs. control sample)
