# Supplementary material for: Evaluating the Effect of Azole Antifungal Agents on the Stress Response and Nanomechanical Surface Properties of Ochrobactrum anthropi Aspcl2.2
Source: Molecules. 2020 Jul 23;25(15):3348. doi: 10.3390/molecules25153348 (PMC7435821; doi:10.3390/molecules25153348)
Supplement: Supplementary file 1 [file molecules-25-03348-s001.zip › Table S1.docx]

Table S1. Summarizing table - the effects of each of the azoles on measured cell parameters.

|  | Parameter | Fc | Ep | Cb | Cl |
| --- | --- | --- | --- | --- | --- |
| Oxidative stress response | GSSG + GSH | ↑↑ | ↑↑ | ns | ↑↑ |
|  | GSTs | ↑↑ | ns | ↑↑ | ↓ |
| Cell texture | Ra | ns | ns | ↓↓ | ns |
|  | Rq | ns | ns | ↓↓ | ns |
|  | R3z | ns | ns | ↓↓ | ns |
| Cell adhesion | Adhesion energy | ↑↑↑↑ | ↑↑↑↑ | ↑↑↑↑ | ↑↑↑↑ |
|  | Adhesion force | ↑↑↑ | ↑↑ | ↑↑↑ | ↑↑ |
|  | Cell surface hydrophobicity | ns | ↓↓ | ↓↓ | ↓↓↓↓ |
| Elastic properties | Deformation | ↑↑↑↑ | ↑↑↑ | ↑↑↑ | ↑↑↑↑ |
|  | Young’s modulus | ↓↓↓↓ | ↓↓↓ | ↓↓↓↓ | ↓↓↓↓ |
|  | Stiffness | ↓↓↓↓ | ↓↓ | ↓↓↓↓ | ↓↓↓↓ |

GSSG + GSH – the level of total glutathione; GSTs – activity of glutathione S-transferases;
Ra – roughness average; Rq – root mean square roughness; R3z – average roughness depth.

- **ns** – the modification was not significant (vs. control sample)
- the direction of the arrows indicates up-/down-regulation
- the number of the arrows indicates the strengths of the modification:

I – increase/decrease by up to 1.0-fold (vs. control sample)

II – increase/decrease within the range from 1.1 to 2.0-fold (vs. control sample)

III – increase/decrease within the range from 2.1 to 3.0-fold (vs. control sample)

IIII – increase/decrease by at least 3.1-fold (vs. control sample)
