# Supplementary material for: Evaluating the Effect of Azole Antifungal Agents on the Stress Response and Nanomechanical Surface Properties of Ochrobactrum anthropi Aspcl2.2
Source: Molecules. 2020 Jul 23;25(15):3348. doi: 10.3390/molecules25153348 (PMC7435821; doi:10.3390/molecules25153348)

Figure S1. Representative AFM height images showing morphological differences between (a) untreated control cells of *O. anthropi* AspCl2.2; cells exposed to: (b) fluconazole; (c) epoxiconazole; (d) climbazole; (e) clotrimazole.

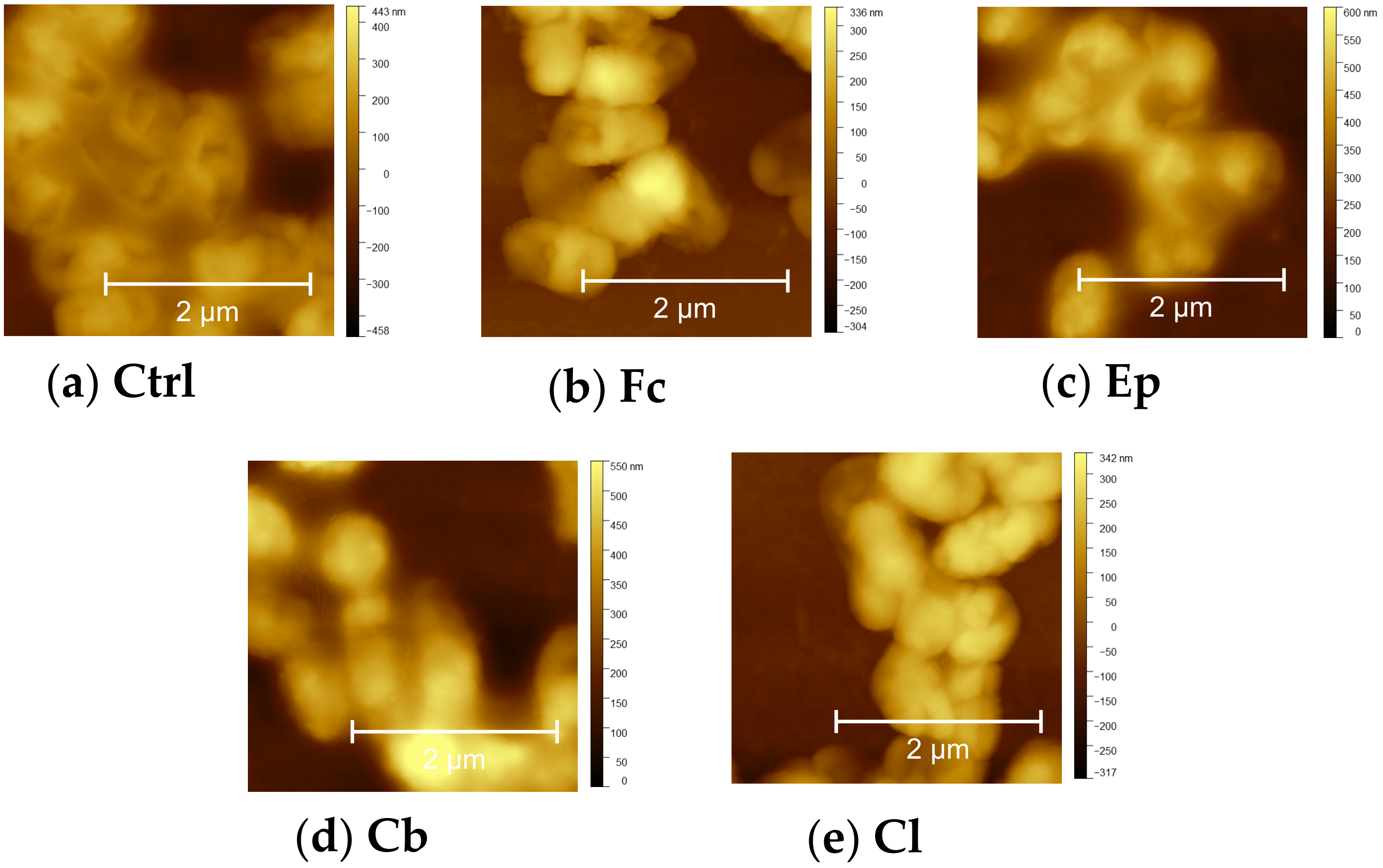

Supplement: Supplementary file 1 [file molecules-25-03348-s001.zip › Figure S1.pdf]
