# Supplementary material for: Evaluating the Effect of Azole Antifungal Agents on the Stress Response and Nanomechanical Surface Properties of Ochrobactrum anthropi Aspcl2.2
Source: Molecules. 2020 Jul 23;25(15):3348. doi: 10.3390/molecules25153348 (PMC7435821; doi:10.3390/molecules25153348)

Appendix 1

Procedure of roughness calculation

Surface roughness was calculated from the single cell images of the same sample (at least fifteen cells of each culture were included in the calculations). Three roughness parameters were chosen as indicative of bacterial cell surface roughness alterations (an average roughness depth, R3z; root mean square roughness, Rq; and roughness average, Ra) and were calculated as follows:

Roughness average (Ra) - the arithmetical average of the absolute values of the height of the surface profile:

$$R_a = \frac{1}{N}\sum_{j=1}^{N}\left|r_j\right|$$

$$r_j = z_j - \bar{z}$$

where: N – the evaluation length; *z* – height of the profile

Root mean square roughness (Rq) – the mean of the measured height deviations within the evaluation length:

$$R_q = \sqrt{\frac{1}{N}\sum_{j=1}^{N} r_j^2}$$

Average roughness depth (R3z) - the distance between the third highest peak and the third lowest valley.

Figure A1. An example of surface roughness determination.

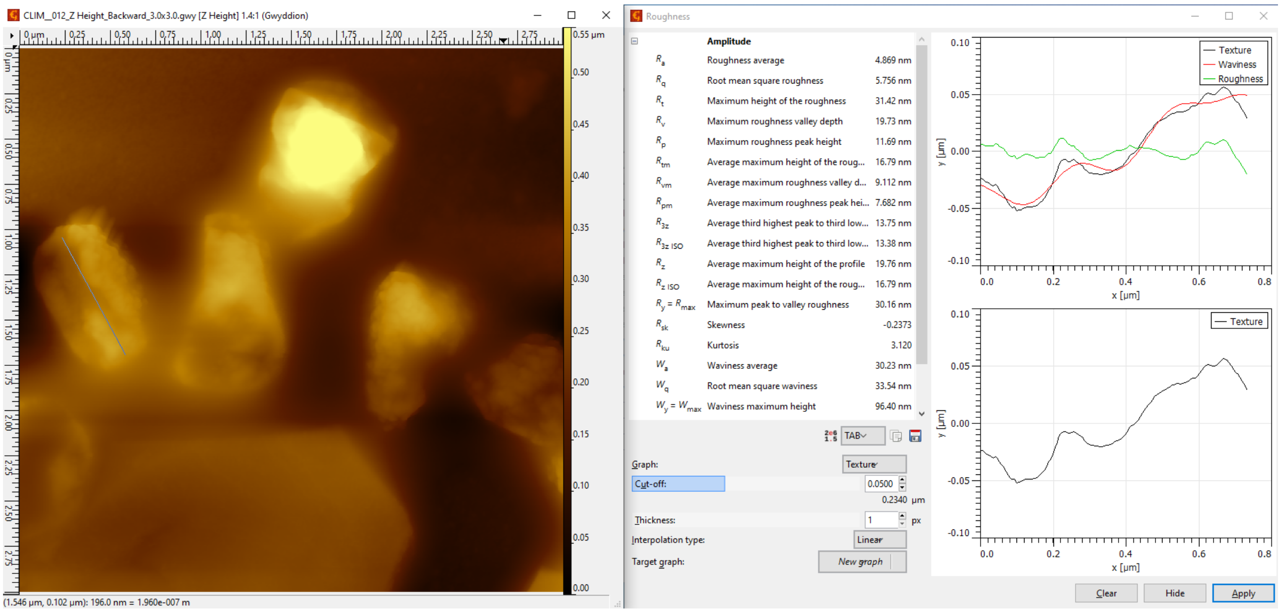

Supplement: Supplementary file 1 [file molecules-25-03348-s001.zip › Appendix 1.pdf]
